# Developing an Inpatient Relationship Centered Communication Curriculum (I-RCCC) rounding framework for surgical teams

**DOI:** 10.1186/s12909-023-04105-7

**Published:** 2023-03-01

**Authors:** Aussama K. Nassar, Barbette Weimer‑Elder, Rachel Yang, Merisa Kline, Bryan K. Dang, David A. Spain, Lisa M Knowlton, Andre B. Valdez, James R. Korndorffer, Tyler Johnson

**Affiliations:** 1 Department of Surgery, Stanford University, Stanford, CA USA; 2 Stanford Medicine Patient Experience, Physician Partnership Team, Stanford, CA USA

**Keywords:** Relationship centered, Communication, Curriculum, Training, Inpatient

## Abstract

**Background:**

Morning rounds by an acute care surgery (ACS) service at a level one trauma center are uniquely demanding, given the fast pace, high acuity, and increased patient volume. These demands notwithstanding, communication remains integral to the success of surgical teams. Yet there are limited published curricula that address trauma inpatient communication needs. Observations at our institution confirmed that the surgical team lacked a shared mental model for communication. We hypothesized that creating a relationship-centered rounding conceptual framework model would enhance the provider-patient experience.

**Study design:**

A mixed-methods approach was used for this study. A multi-pronged needs assessment was conducted. Provider communion items for Press Ganey and Hospital Consumer Assessment of Healthcare Providers and Systems (HCAHPS) surveys were used to measure patients’ expressed needs. Faculty with experience in relationship-centered communication observed morning rounds and documented demonstrated behaviors. A five-hour workshop was designed based on the identified needs. A pre-and post-course Assessment and course evaluation were conducted. Provider-related patient satisfaction items were measured six months before the course and six months after the workshop.

**Results:**

Needs assessment revealed a lack of a shared communication framework and a lack of leadership skills for senior trauma residents. Barriers included: time constraints, patient load, and interruptions during rounds. The curriculum was very well received. The self-reflected behaviors that demonstrated the most dramatic change between the pre and post-workshop surveys were: *I listened without interrupting; I spoke clearly and at a moderate pace; I repeated key points; and I checked that the patient understood.* All these changed from being performed by 50% of respondents “about half of the time” to 100% of them “always”. Press Ganey top box likelihood to recommend (LTR) and provider-related top box items showed a trend towards improvement after implementing the training with a percentage difference of up to 20%.

**Conclusion:**

The Inpatient Relationship Centered Communication Curriculum (I-RCCC) targeting senior residents and Nurse Practitioners (NP) was feasible, practical, and well-received by participants. There was a trend of an increase in LTRs and provider-specific patient satisfaction items. This curriculum will be refined based on the study results and potentially scalable to other surgical specialties.

## Introduction

Surgical residents undergo rigorous training in technical skills acquisition and patient management; however, emphasis on non-technical and practical communication skills is often overlooked [1]. In addition, surgical residents face numerous communication obstacles due to limited time, frequent interruptions, and a lack of a shared framework for communicating with team members and patients [2]. Given these constraints, efficiently achieving optimal outcomes is a challenge. Nevertheless, communication is a crucial skill required for the interaction of any two people. Communication between provider and patient is fundamental and has changed over the years from provider-centric to patient-centric and relationship-centered [3]. The Academy of Communication in Healthcare has proposed a relationship-centered care model as an optimal communication framework. Relationship centered care (RCC) in healthcare is based on four principles: [1] relationships in healthcare should include the personhood of the participants (both patient and health care providers), [2] participants’ affect and emotions are essential components of these relationships, [3] all health care relationships occur within the context of reciprocal influence, and [4] formation and maintenance of genuine relationships in health care are morally valuable [4].

RCC can have a transformative effect on patient care and provider wellness [3]. When team members prioritize relationships with patients, families, and other team members, all parties benefit [3, 5].

Surgical care on inpatient units at teaching hospitals is typically provided via early morning rounding with house staff, Advanced Practice Providers (APPs): nurse practitioners (NPs), physician assistants (PAs), nursing staff, and physician attendings. These rounds function most effectively when the patient’s opinions and every team member are heard and valued [5]. These skills are even more important in episodic, fast-paced, interactive areas such as the Emergency Room or procedural areas because time is limited. Listening to the patient’s concerns results in more effective and efficient encounters as a provider develops more meaningful interpersonal connections with the patient. Developing these relationships also increases professional fulfillment and mitigates burnout [6]. Nevertheless, the challenge remains: how can busy surgical services implement an optimal communication model in an environment where providers are stressed and time is limited? To our knowledge, no similar published curricula address those needs.

We hypothesized that creating a curriculum built from RCC principles would help create a shared communication framework to improve rounding efficiency, develop consistency, and enhance patients’ experience.

## Methods

To create a relationship-centered culture at our institution, a Physician Partnership Program was formed with physicians and a patient experience team who have expertise in communication. In 2015, this program implemented a suite of strategic initiatives aimed at facilitating the adoption of a shared communication model. The focus was on providing evidence-based relationship-centered skills to clinicians to foster a shared mental model for communication across all specialties. Identifying faculty champions to facilitate fundamental relationship-centered communication skills was a critical initial step. To train the faculty champions, a partnership with the Academy on Communication in Healthcare (ACH) was formed in 2016 to deliver high-quality content and facilitate skill-building and feedback. The Acute Care Surgery (ACS) and Trauma team partnered with the patient experience team to develop a new curriculum aimed at that goal. The curriculum focused on provider-specific skills related to communication. The senior resident and APPs were enrolled in the initial study because they play a crucial role in clinical leadership and have the most “face time” with patients on the floor. The team piloted this curriculum by designing a workshop for Trauma/ACS APPs and senior residents.

**Fig. 1:**
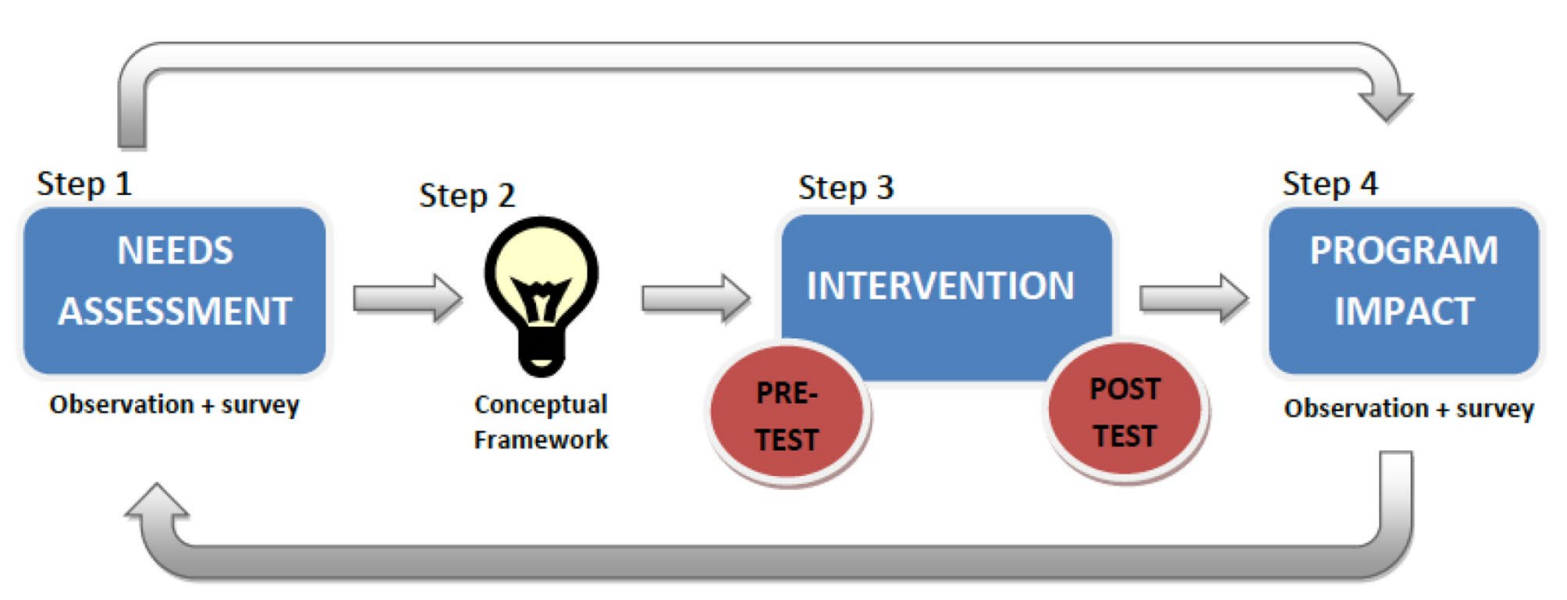
I-RCCC Study Design. Schematic Diagram illustrating our study design that comprises of 4 steps. Step 1 is a needs assessment analysis that included both observed needs (by communication experts) and assessed needs using an electronic survey. Step 2 is developing the I-RCCC conceptual framework from the data collected in Step 1. The intervention (step 3) is the implementation of the I-RCCC. Evaluation is carried out before and after the course. The last step (step 4) is assessing program impact through both observations as well as patient satisfaction surveys to assess the strengths and limitations of the I-RCCC. Our study uses an evidence-based model for program improvement. It relies on the constant refinement of our curriculum from trainee and patient feedback

Approval from the Institutional Review Board was obtained from Stanford University School of Medicine. Our study design utilized an evidence-based model for program improvement [7]. It relies on the constant refinement of our curriculum from trainee and patient feedback (Fig. 1). This model includes four steps: an initial need-based assessment, design, implementation, and evaluation.

### Analysis- needs assessment

To develop the pilot curriculum, we began assessing needs using a mixed-methods approach (Fig. 1). This included:

(1) Before implementing this initiative, the hospital Consumer Assessment of Healthcare Providers and Systems (HCAHPS) survey and Press Ganey results from 6 months were collected. Particular attention was paid to the provider-specific items related to communication. From the Press Ganey scale, these items were: (a) physician kept you informed, (b) physician response to concerns and complaints, and (c) physician concern questions/worries;

(2) Communication experts from the Physician Partnership Program and Stanford University directly observed morning rounds on the trauma service; and.

(3) healthcare providers’ (physicians, APPs, and physician attendings) perceived needs were explored via an electronic survey.

### I-RCCC design and implementation

We then used the needs assessment data to develop a conceptual framework for the I-RCC curriculum (Fig. 1). The curriculum highlighted four modules: [1] listening to connect, [2] expecting emotions, [3] eliciting concerns, and [4] summarizing the plan. The participants were fourth-year postgraduate (PGY4) residents and APPs.

**Fig. 2:**
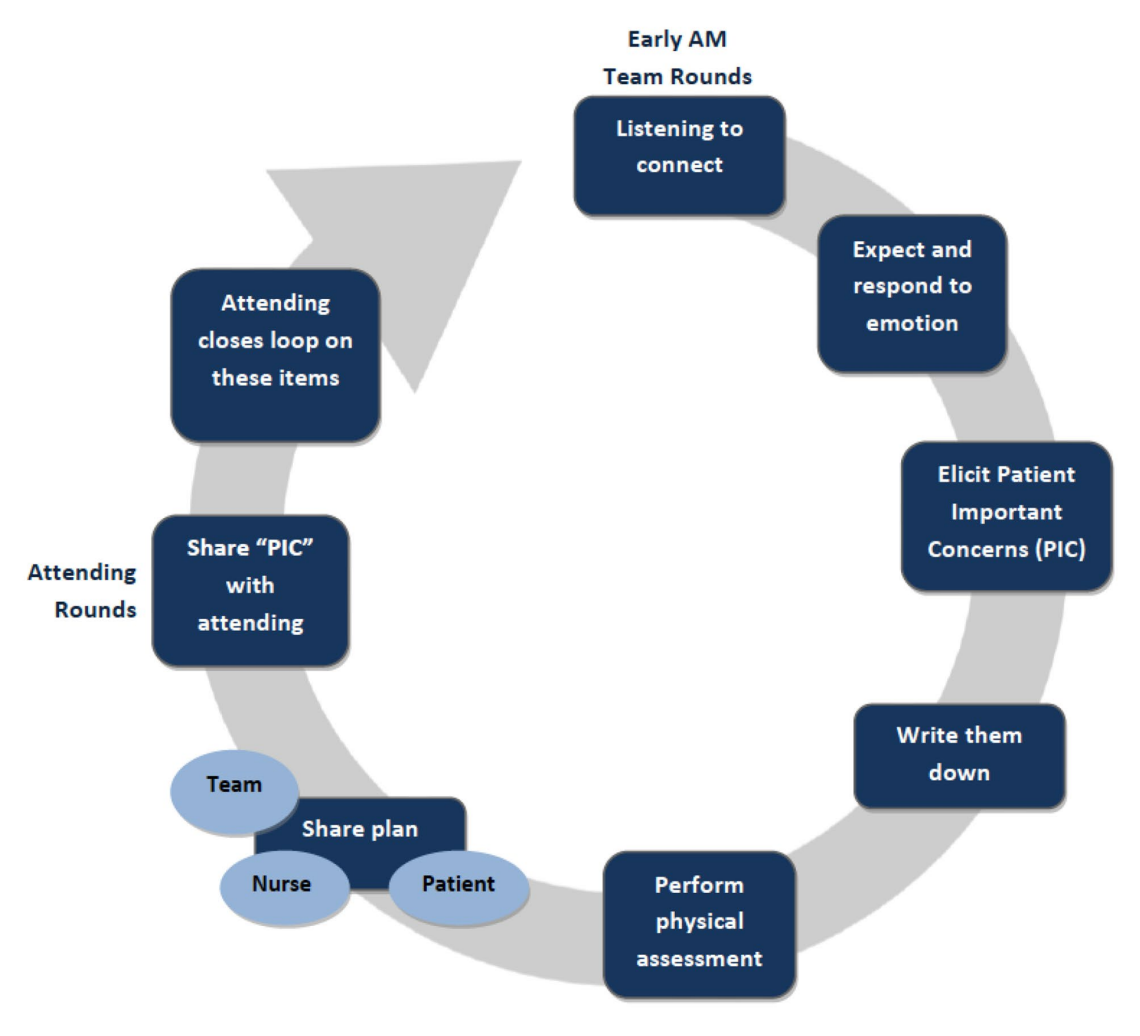
Inpatient Relationship-Centered Communication Rounding Process. Schematic diagram outlining the I-RCCC rounding process implemented in our study. The process is based on our four modules: listening to connect, expecting emotions, eliciting concerns, and summarizing the plan

Each workshop module consisted of a rounding process to support the modules was outlined at the start of the course (Fig. 2), For each discrete skill there was a didactic component, followed by a live or video demo to imprint positive behavior and to illustrate the new rounding framework. We pre-recorded a typical encounter on the trauma unit with a trained, standardized patient. We then provided the participants with script of a typical encounter. An example of scripts: “ Good morning, how are you feeling today”, Your concerns are important to us. What can we do for you today?” Exhaustive “What else?’, “Our plan today to address your concerns and ours is. . .”. This was followed by peer-to-peer role play to apply key communication concepts and responses to patient emotions were also practiced: participants were assigned the role of a clinician or patient and then read the script verbatim aloud. The workshop’s final portion was an integrated exercise that allowed each participant to practice these skills in a rounding simulation with multiple patients.

### Evaluation

The evaluation was carried out before and after course implementation via pre and post-course reflective assessment and overall course evaluation (Fig. 1). To ensure and aid in implementing the framework, coaching of trainees and a series of meetings with nursing staff were performed throughout the following weeks to establish behavior change. Descriptive statistics were used. HCAHPS and Press Ganey survey results from six months before course implementation compared to results from six months post. Due to the sample size, descriptive statistics were utilized to show trends. The patients’ satisfaction surveys were designed to ask patients about their overall hospital experience, including their experience with the attending/consultant physician providers. We opted to target the senior residents and APPs on our Acute Care Surgery Service/Trauma service (for the pilot phase) for the following reasons: 1)Due to the emergency nature of the work on the Acute Care Surgery/Trauma service the week rounding Attending/Consultant won’t be able to see all of the patients every day 2)Morning rounds and subsequent patient touchpoints throughout the day on the acute care surgery service are usually led by the senior residents and APPs. Since most of the patient’s face-to-face contact is with the senior residents and APPs we thought that by targeting them we would get the highest impact on patient experience. 3) By training the senior residents and APPs, they would role model a positive behavior to other team members.

## Results

### Needs assessment- observed needs

The observed needs assessment performed by two communication experts who directly observed morning rounds on multiple occasions revealed a lack of a shared communication framework for the team and a lack of leadership skills for senior trauma residents. This observed needs assessment was based on experts’ opinions of communication and leadership skills on morning rounds.

### Needs assessment- expressed needs (survey)

The trauma providers expressed needs survey (Table 1), its participants consisted of 5 physician attendings, 23 residents, 3 NPs, and two others (total of 33 respondents). Most physician attendings (80%) have stated participation in prior communication skills training compared to 31% of the residents and 0% of interns. In terms of course delivery. The majority of participants favored an online method: 80% of physician attendings, 50% of residents, 71% of interns, and 67% of NPs. Followed by small-group discussion (25% residents, 29% interns) and simulated and real-life experiences (20% physician attendings, 6% residents). Regarding communication needs, most respondents’ goal from a communication skills curriculum was to improve their own communication skills (80% physician attendings, 69% residents, and all interns), followed by “I need to improve my patients’ experience” (Table 1).

**Table 1:** Summary of needs assessment responses by item and respondent title

	Attending	PGY	Intern	APP	Other	Total
**Item** (%)	**(n = 5)**	**(n = 16)**	**(n = 7)**	**(n = 3)**	**(n = 2)**	**(n = 33)**
**Preferred method of course delivery**						
Online	80	50	71	67	0	58
Didactic lectures	0	6	0	33	0	6
Small-group discussion	0	25	29	0	50	21
Self-reflection	0	6	0	0	0	3
Role play	0	0	0	0	0	0
Simulated and real-life experiences	20	6	0	0	50	9
Mix of online and in person	0	0	0	0	0	0
**Current communication needs**						
I want to improve my communication skills	80	69	100	67	100	79
I want to help others improve their communication skills	60	50	29	0	50	43
I need to improve my patients’ experience	80	44	43	67	50	52
**Familiarity with RCC**						
I am not at all familiar	20	56	29	33	100	45
I am somewhat familiar	60	38	71	67	0	49
I practice RCC	20	0	0	0	0	3
**Communication skills you want to improve**						
Creating rapport	40	31	14	33	0	27
Eliciting all concerns	100	38	57	67	100	58
Negotiating the agenda	20	44	43	100	0	43
Opening the conversation	20	13	0	0	0	9
Exploring perspectives and naming emotions	20	19	14	0	50	18
Responding with compassion	40	19	0	0	0	15
Sharing information	20	38	57	0	100	40
Assessing understanding	20	38	71	33	100	46
Summarizing and clarifying	0	25	43	33	0	24
Conflict management	60	63	71	67	100	67
Clinician-clinician communication	60	44	71	33	0	48
Handoffs with team members	20	44	86	33	0	46
**I quickly establish rapport**						
Almost always/usually	100	75	86	100	100	85
occasionally/rarely	0	25	14	0	0	15
**I consistently elicit patients’ concerns**						
Almost always/usually	40	38	43	33	0	37
Occasionally/rarely	60	62	57	67	100	63
**I negotiate with patients to establish the agenda**						
Almost always/usually	20	50	29	67	50	43
Occasionally/rarely	80	50	71	33	50	57
**I use open-ended questions to explore patients’ perspectives**						
Almost always/usually	60	50	71	67	50	58
Occasionally/rarely	40	50	29	33	50	42
**I offer opportunities for patients to express emotions**						
Almost always/usually	60	69	71	67	50	67
Occasionally/rarely	40	31	29	33	50	33
**I acknowledge patients’ emotions with empathic responses**						
Almost always/usually	60	81	86	100	100	82
Occasionally/rarely	40	19	14	0	0	18
**I share information in small “chunks"**						
Almost always/usually	60	86	57	100	50	75
Occasionally/rarely	40	14	43	0	50	25
**I consistently check my patients’ understanding**						
Almost always/usually	60	69	57	100	50	67
Occasionally/rarely	40	31	43	0	50	33
**I encourage my patients to summarize what we discussed**						
Almost always/usually	40	31	14	33	50	30
Occasionally/rarely	60	69	86	67	50	70
**I regard communication training as being relevant**						
Almost always/usually	100	94	100	100	50	94
Occasionally/rarely	0	6	0	0	50	6
**Quantity of work on rounds promotes communication**						
Agree/strongly agree	0	6	0	33	0	6
Disagree/strongly disagree	80	94	100	67	100	91
**Morning rounds are calm/easeful**						
Agree/strongly agree	0	6	14	67	0	12
Disagree/strongly disagree	80	94	86	33	100	85
**I often feel rushed on morning rounds**						
Agree/strongly agree	80	94	71	67	100	85
Disagree/strongly disagree	0	6	29	33	0	12
**There is sufficient time to address patients’ concerns**						
Agree/strongly agree	20	12	14	0	0	12
Disagree/strongly disagree	60	88	86	100	100	85
**There are multiple interruptions most days**						
Agree/strongly agree	60	81	71	67	50	73
Disagree/strongly disagree	40	19	29	33	50	27
**There is adequate time to obtain a treatment plan**						
Agree/strongly agree	40	31	29	33	100	36
Disagree/strongly disagree	40	69	71	67	0	61
**EMR documentation allows for optimal communication**						
Agree/strongly agree	0	6	0	33	50	9
Disagree/strongly disagree	80	94	100	67	50	88
**Having bedside nurses makes rounds more efficient**						
Agree/strongly agree	80	56	57	100	0	60
Disagree/strongly disagree	0	44	43	0	100	37
**Non-English speaking patients are easily accommodated**						
Agree/strongly agree	20	0	14	33	0	9
Disagree/strongly disagree	60	100	86	67	100	88
**Trauma service is one of the busiest services**						
Agree/strongly agree	60	88	71	67	50	76
Disagree/strongly disagree	20	12	29	33	50	21
**My main goal on rounds is to gather necessary information**						
Agree/strongly agree	20	50	54	0	0	39
Disagree/strongly disagree	60	50	46	100	100	58
**We treat our trauma patients with courtesy and respect**						
Agree/strongly agree	60	88	43	100	100	76
Disagree/strongly disagree	20	12	57	0	0	21
**Trauma service does a great job of listening to patients**						
Agree/strongly agree	20	25	29	100	50	33
Disagree/strongly disagree	60	75	71	0	50	64
**We give explanations understandable to patients**						
Agree/strongly agree	40	50	14	100	50	45
Disagree/strongly disagree	40	50	86	0	50	52
**We explain new medications and side effects every time**						
Agree/strongly agree	0	6	0	33	0	6
Disagree/strongly disagree	80	94	100	67	100	91
**We explain new tests/imaging every time**						
Agree/strongly agree	20	44	29	67	0	37
Disagree/strongly disagree	60	56	71	33	100	60

PGY: Post -graduate year

APPs: Advanced Practice Providers

When surveyed about the communication skills they might want to improve, 100% of physician attendings chose “eliciting all concerns of patients,“ while 63% requested “conflict management,“ followed by “eliciting patients’ concerns” (44%). On the other hand, interns chose conflict management (71%) and clinician-clinician communication (71%). All NPs requested training on “negotiating the agenda” followed by “conflict resolution” (Table 1). Regarding perceived barriers to effective communications on the trauma floor, most respondents strongly agreed that the quantity of work interferes with effective communication. They also believed that morning rounds were rushed with multiple interruptions. In addition, the majority felt that non-English speaking patients were not accommodated. Respondents also believed that having bedside nurses present on rounds makes rounds more efficient. Furthermore, they felt that the trauma/ACS service does an inadequate job of listening to patients, explaining new medications, side effects, and new tests/imaging.

### I-RCCC course survey

With respect to the pre and post-pilot course survey results, there were 7 participants (3 PGY 4 residents and 4 NPs) (Table 2). The self-reflected behaviors that demonstrated the most dramatic change between the pre and post-workshop surveys were: *I listened without interrupting; I spoke clearly and at a moderate pace; I repeated key points; and I checked that the patient understood.* All these changed from being performed by 50% of respondents “about half of the time” to 100% of them “always”.

**Table 2:** Summary of pre- and post- course survey responses by item and respondent title

	Pre Course		Post Course	
	PGY4	APP	PGY4	APP
Item (%)	(*n* = 2)	(*n* = 4)	(*n* = 3)	(*n* = 4)
**I greeted the patient with a kind attitude**				
Always/most of the time	100	100	100	100
About half the time/sometimes/never	0	0	0	0
**I maintained appropriate eye contact**				
Always/most of the time	100	75	100	100
About half the time/sometimes/never	0	25	0	0
I **listened without interrupting**				
Always/most of the time	50	75	100	100
About half the time/sometimes/never	50	25	0	0
**I encouraged the patient to voice concerns**				
Always/most of the time	100	75	100	100
About half the time/sometimes/never	0	25	0	0
**I spoke clearly and at a moderate pace**				
Always/most of the time	50	100	100	100
About half the time/sometimes/never	50	0	0	0
**I used non-medical language**				
Always/most of the time	100	100	100	100
About half the time/sometimes/never	0	0	0	0
**I limited discussion to fewer than 5 key points**				
Always/most of the time	100	75	100	100
About half the time/sometimes/never	0	25	0	0
**I gave specific, concrete explanations**				
Always/most of the time	50	50	100	100
About half the time/sometimes/never	50	50	0	0
**I repeated key points**				
Always/most of the time	50	75	100	100
About half the time/sometimes/never	50	25	0	0
**I used graphics to help explain something**				
Always/most of the time	0	0	100	50
About half the time/sometimes/never	100	100	0	50
**I asked the patient what questions he/she had**				
Always/most of the time	100	100	100	100
About half the time/sometimes/never	0	0	0	0
**I checked that the patient understood**				
Always/most of the time	50	50	100	100
About half the time/sometimes/never	50	50	0	0

Course evaluation results collected at the end of the workshop showed that 100% of residents and 75% of NPs would recommend this workshop, while 67% of residents and 100% of NPs thought it was extremely/very effective (Table 3).

**Table 3:** Summary of course evaluation responses by item and respondent title

Item (%)	PGY4 (n = 3)	APP (n = 3)
**How helpful was the workshop content**		
Extremely helpful	67	50
Very helpful	0	25
Somewhat helpful	33	0
Not too helpful	0	0
Not at all helpful	0	0
**How you would rate the overall workshop**		
Excellent	67	75
Good	0	0
Average	33	0
Poor	0	0
Very poor	0	0
**Would you recommend this workshop**		
Yes	100	75
No	0	0
**Describing the 4 principles of RCC**		
Extremely effective	67	100
Somewhat/not at all effective	33	0
**Assessing current knowledge of the 4 principles**		
Extremely effective	67	100
Somewhat/not at all effective	33	0
**Describing strategies to respond to patients’ emotion**		
Extremely effective	100	100
Somewhat/not at all effective	0	0
**Practicing eliciting the patient’s concerns**		
Extremely effective	67	100
Somewhat/not at all effective	33	0
**Discussing the RCR process**		
Extremely effective	100	100
Somewhat/not at all effective	0	0
**Practicing RCR in a simulated setting**		
Extremely effective	67	100
Somewhat/not at all effective	33	0
**Anything that could make it challenging to apply what you learned**		
Yes	100	75
No	0	0

### Patient satisfaction survey

**Fig. 3:**
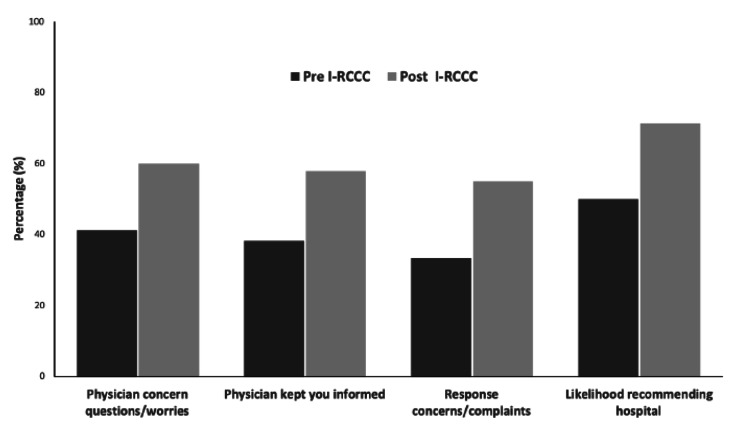
Patient Satisfaction Survey Results Pre and Post I-RCCC Course Implementation. Graphical representation of patient Press Ganey/HCAHPS scores for the three provider-related elements and LTR compared 6-months pre- and post I-RCCC implementation. Pre-IRCCC n = 34 and Post-IRCCC n = 20

One of our study’s primary outcomes is its ability to improve patient experience, as demonstrated by the Press Ganey survey. The three provider-related elements’ Press Ganey scores improved when comparing the six months pre- and six months post I-RCCC implementation (Fig. 3). In addition, all three provider-related elements and the LTR top box scores on the Press Ganey survey revealed an upward trend after implementing the I-RCCC course (Fig. 3). While the intervention lacks a control group, we are unaware of any other major initiative or change during this time.

## Discussion

The importance of communication in healthcare is not just intuitive but backed up by evidence. At our institution, patient experience data demonstrated an opportunity for improvement for the ACS/Trauma Service. Literature indicates that communication behaviors can be learned via structured didactic exercises that include role-play [8, 9]. A few studies looked at the impact of rounding on improved patient satisfaction [10], patient teaching, and less adverse events were cited in the beneficial outcomes of relationship-centered care. Our team created a meaningful, comprehensive, practical, and efficient method for disseminating communication skills to Stanford surgery residents and NPs. Through cycles of evaluation and refinement, we restructured the workshop to reflect the ongoing needs of the healthcare team (Fig. 1).

### Needs assessment

The needs assessment demonstrated that patients felt communication skills needed improvement in some important care provider domains. Further, direct observations from Stanford communication experts identified a need for a shared communication model and room for improving communication skills. Finally, surveys of residents likewise demonstrated that residents understood the importance of communication generally and their need to improve their own communication skills (Table 1).

### I-RCCC

The workshop and related curriculum were developed in response to this need. Although the skills needed to be a competent clinician are widely agreed upon and set by the American Council of Graduate Medical Education (Stanford Graduate Medical Education 2019), effective educational methods to teach these skills and the impact of such educational tools have been lacking (11). Therefore, the I-RCCC was designed to provide deliberate observation, feedback, and self-reflection to gain insight into trainees’ communication behaviors.

Our workshop consisted of a pedagogically consistent series of four modules that can be easily taught over a half-day workshop. The curriculum presents educational points that address the concerns of patients and providers. The teaching workshop teaches, demonstrates, and allows residents to experience important communication behaviors. Surveys of residents demonstrated that they viewed the intervention highly and would universally recommend it to their peers (Table 3). Furthermore, before and after videos of senior residents in a common communication scenario demonstrated that they were significantly and consistently more likely to implement core communication behaviors after the intervention.

### I-RCCC strengths

One of our study’s main strengths is the rigorous approach to assessing needs before designing the intervention, which assured that residents would be invested in the teaching and approach. Moreover, the workshop is feasible and could be implemented similarly in surgical residencies nationally. In addition, the intervention was designed in part by surgeons and thus reflected a real-world understanding of the reality of surgical wards. Resident feedback also demonstrated that they appreciated the class and thought it was effective (Table 3). Lastly, the workshop addressed a pressing need and was easily deployable.

### I-RCCC limitations

This study’s limitations include that it was conducted at a single institution with only one cohort of senior residents and NPs. However, as this study was exploratory in nature, we have now been able to streamline a process whereby it can be expanded to additional healthcare providers at all levels and maybe to other institutions. The evidence for the curriculum’s efficacy remains limited, given that we need long-term patient satisfaction data. Furthermore, any skills gained during this training may decay over time. Likewise, while we designed this to be a real-world, implementable curriculum, it is nonetheless logistically challenging to obtain 100% attendance of all residents for the required training. Also, there were 34 Press Ganey patient surveys in the pre and 21 in the post-survey; this difference in the total number of returned surveys (although showing a positive trend) would argue against the significance of the results. In addition, this is a self-reported communication behavior, and the patient response data bolster the program’s effectiveness.

### Next steps

We plan to further develop and implement I-RCCC-2.0 and assess for improved outcomes. Our pilot curriculum allowed us to explore the utility and feasibility of a new model for relationship-centered-rounding. This pilot study informed us of the challenges of implementing such a model on a busy trauma surgery service and the perceived barriers by participating practitioners. We hope our revised curriculum will prove to be even more beneficial to our patients and that future studies will demonstrate this. In addition, to address skill decay, we launched our Stanford communication coaching program shortly after the implementation of I-RCCC. With the coaching program each resident is assigned to a qualified faculty coach that provides longitudinal ongoing targeted feedback at the point of care. Stanford coaching program impact is currently being evaluated, and results will be published in the near future.

## Conclusion

Using a rigorous and multi-faceted needs assessment, we pioneered a novel, efficient, and effective curriculum for organizing inpatient communication structures in an inpatient trauma surgery service and teaching these behaviors to surgery residents. Furthermore, we demonstrated that these objectives could be effectively implemented in a real-world academic medical institution. The gold standard would be demonstrating that the curriculum improves patient outcomes, though we recognize the inherent challenges of demonstrating this causal relationship. Our future work involves furthering the generalizability of our findings across multiple centers and groups of trainees and emphasizing the reproducibility and importance of the curriculum across clinical settings.

## Data Availability

The datasets generated and/or analyzed during the current study are not publicly available due to the small sample size and the possibility that it might be traced back to respondents. We informed the participants that data will be presented as an aggregate, but it is available from the corresponding author at a reasonable request. We can share the data upon request. Due to the small sample size and the possibility that it might be traced back to respondents, we have informed the participants that data will be presented as an aggregate.

## Abbreviations

ACH: Academy of Communication in Healthcare.
ACS: Acute Care Surgery.
APP: Advanced Care Providers.
HCAHPS: Hospital Consumer Assessment of Healthcare Providers and Systems.
I-RCCC: INPATIENT RELATIONSHIP-CENTERED COMMUNICATION CURRICULUM.
LTR: Likelihood To Recommend.
NP: Nurse Practitioner.
PA: Physician Assistant.
RCC: Relationship Centered Care.
